# Preoperative platelet lymphocyte ratio (PLR) is superior to neutrophil lymphocyte ratio (NLR) as a predictive factor in patients with esophageal squamous cell carcinoma

**DOI:** 10.1186/1477-7819-12-58

**Published:** 2014-03-19

**Authors:** Ji-Feng Feng, Ying Huang, Qi-Xun Chen

**Affiliations:** 1 Department of Thoracic Surgery, Zhejiang Cancer Hospital, No.38 Guangji Road, Banshan Bridge, Hangzhou 310022, China; 2 Key Laboratory Diagnosis and Treatment Technology on Thoracic Oncology, Hangzhou 310022, Zhejiang province, China; 3 Department of Nursing, Zhejiang Cancer Hospital, Hangzhou, China

**Keywords:** Esophageal cancer, Neutrophil lymphocyte ratio, Platelet lymphocyte ratio, Prognostic factor, Squamous cell carcinoma, Survival

## Abstract

**Background:**

Recent studies have shown that the presence of systemic inflammation correlates with poor survival in various cancers. The aim of this study was to determinate the prognostic value of the neutrophil lymphocyte ratio (NLR) and the platelet lymphocyte ratio (PLR) in patients with esophageal squamous cell carcinoma (ESCC).

**Methods:**

Preoperative NLR and PLR were evaluated in 483 patients undergoing esophagectomy for ESCC from January 2005 to December 2008. The prognostic significance of both markers was then determined by both uni- and multivariate analytical methods. Receiver operating characteristic (ROC) curves were also plotted to verify the accuracy of NLR and PLR for survival prediction.

**Results:**

High preoperative NLR (≥3.5 *versus* < 3.5, *P* = 0.039) and PLR (≥150 *versus* < 150, *P* < 0.001) were significantly associated with poor overall survival in multivariate analysis. However, our study demonstrated a better discrimination for the PLR in terms of hazard ratio(HR) than the NLR (HR = 1.840 *versus* HR = 1.339). Patients with NLR ≥3.5 had significantly poorer overall survival compared to NLR <3.5 (35.4% *versus* 57.7%, *P* < 0.001). Patients with PLR ≥150 also had significantly poorer overall survival compared to patients with PLR <150 (32.7% *versus* 63.5%, *P* < 0.001). The area under the curve (AUC) was 0.658 (95% confidence interval (CI): 0.610 to 0.706, *P* < 0.001) for NLR and 0.708 (95% CI: 0.662 to 0.754, *P* < 0.001) for PLR, indicating that PLR was superior to NLR as a predictive factor in ESCC.

**Conclusions:**

Preoperative NLR and PLR were significant predictors of overall survival in patients with ESCC. However, PLR is superior to NLR as a predictive factor in patients with ESCC.

## Background

Esophageal cancer (EC) is the eighth most common type of cancer worldwide. In China, the crude mortality rate of EC in 2005 was 15.2/100,000, which represented 11.2% of all cancer deaths and ranked as the fourth most common cause of cancer death [1]. Although advances have occurred in its multidisciplinary treatment, surgical resection remains the modality of choice [2]. The overall five-year survival after surgical resection is poor; the reason for that is the relatively late stage of diagnosis and rapid clinical progression [3,4]. Therefore, assessing the prognostic factors in EC will become more and more important.

Over the past few decades, a number of prognostic factors for EC have been identified, including depth of invasion, lymph node metastasis, TNM stage and other miscellaneous factors [5,6]. Recently, there is increasing evidence that a systemic inflammatory response is of prognostic value in various cancers [7,8]. C-reactive protein is one index of systemic inflammation. However, C-reactive protein is not routinely measured as part of the preoperative examination. The neutrophil to lymphocyte ratio (NLR) and platelet to lymphocyte ratio (PLR) are other markers, and some studies have shown elevated NLR or PLR to be a significant prognostic factor in cancers [9-11]. However, few studies regarding NLR and PLR in EC are available, and their rolesare still controversial.

Esophageal squamous cell carcinoma (ESCC) is the most common pathological type of EC in China, in contrast to the predominance of adenocarcinoma in the Western world [12]. Thus, the aim of this study was to determine the prognostic value of NLR and PLR in ESCC.

## Methods

### Patients

A retrospective analysis was conducted of 483 patients with ESCC who underwent curative esophagectomy in our department from January 2005 to December 2008. All of the patients included in the analysis fit the criteria: (1) ESCC confirmed by histopathology; (2) curative esophagectomy with R0 resection (*en bloc* resection with margins histologically free of disease); (3) at least six lymph nodes examined for pathological diagnosis; (4) surgery neither preceded nor followed by adjuvant chemotherapy and/or radiotherapy; and (5) preoperative NLR and PLR obtained before esophagectomy. All subjects gave written informed consent to the study protocol, which was approved by the Ethical Committees of Zhejiang Cancer Hospital, Hangzhou, China.

In our institute, patients were followed up at our outpatient department every three to six months for the first two years after resection, then annually. Recording of medical history, physical examination and computed tomography of the chest were performed during the follow-up. Endoscopy was obtained in cases of clinically indicated recurrence or metastasis. The last follow-up was 30 November 2011.

### Surgery

The standard surgical approach consisted of a limited thoracotomy on the right side and intrathoracic gastric reconstruction (Ivor Lewis procedure) for lesions at the middle/lower third of the esophagus. Upper third lesions were treated by cervical anastomosis (McKeown procedure). In our institute, two types of lymphadenectomy were carried out as a standard procedure. The majority of patients underwent two-field lymphadenectomy. In this cohort of patients, thoracoabdominal lymphadenectomy was performed, including the subcarinal, paraesophageal, pulmonary ligament, diaphragmatic and paracardial lymph nodes, as well as those located along the lesser gastric curvature, the origin of the left gastric artery, the celiac trunk, the common hepatic artery and the splenic artery. Three-field lymphadenectomy was performed only if the cervical lymph nodes were thought to be abnormal upon preoperative evaluation.

### NLR and PLR evaluation

Data on preoperative blood cell counts were extracted in a retrospective fashion from the medical records. All white blood cell and differential counts were taken within one week prior to surgery. The NLR was defined as the absolute neutrophil count divided by the absolute lymphocyte count, and it was categorized into two groups [13] (<3.5 and ≥3.5); similarly, PLR was defined as the absolute platelet count divided by the absolute lymphocyte count, and it was also categorized into two groups [10,11] (<150 and ≥150).

### Statistical analysis

Statistical analysis was conducted with SPSS 17.0 (SPSS Inc., Chicago, IL, USA). The Pearson Chi squared test was used to determine the significance of differences for patients grouped by NLR and PLR. The overall cumulative probability of survival was calculated by the Kaplan-Meier method, and the difference was assessed by the log-rank test. Univariateand multivariate analyses of Cox regression proportional hazard model were performed to evaluate the prognostic parameters for overall survival with the enter method. HRs with 95% CIswere used to quantify the strength of the association between predictors and survival. ROC curves were also plotted to verify the accuracy of NLR and PLR for overall survival prediction. A *P* value less than 0.05 was considered statistically significant.

## Results

### Patient characteristics

**Figure 1:**
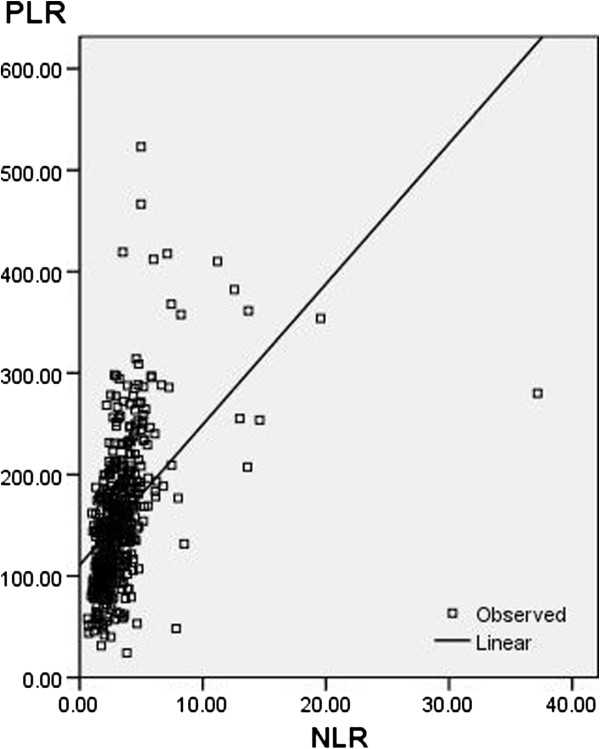
**Correlation between the NLR and PLR (r = 0.483, *P*** **< 0.001).** NLR, neutrophil lymphocyte ratio; PLR platelet lymphocyte ratio.

Among the 483 patients, 72 (14.9%) were women and 411 (85.1%) were men. The mean age was 59.1 ± 8.0 years, with an age range from 34 to 80 years. All the clinicopathologic characteristics were comparable between patients grouped by NLR or PLR, as shown in Table 1. Our study showed that NLR or PLR was associated with tumor size, differentiation, depth of invasion and nodal metastasis. In addition, there was a positive correlation between the NLR and PLR (r = 0.483, *P* < 0.001) (Figure 1).

**Table 1:** The characteristics of the 483 patients grouped by NLR and PLR

	**Cases (number, %)**	**NLR (number)**		***P *** **value**	**PLR (number)**		***P *** **value**
		**<3.5**	**≥3.5**		**<150**	**≥150**	
Gender				0.143			0.473
Female	72 (14.9)	51	21		42	30	
Male	411 (85.1)	254	157		221	190	
Age (years)				0.305			0.804
≤60	273 (56.5)	167	106		150	123	
>60	210 (43.5)	138	72		113	97	
Tumor length				<0.001			<0.001
≤3	138 (28.6)	117	21		95	43	
>3	345 (71.4)	188	157		168	177	
Tumor location				0.101			0.561
Upper	27 (5.6)	12	15		15	12	
Middle	247 (51.1)	156	91		140	107	
Lower	209 (43.3)	137	72		108	101	
Vessel involvement				0.121			0.729
Negative	407 (84.3)	263	144		223	184	
Positive	76 (15.7)	42	34		40	36	
Perineural invasion				0.862			0.882
Negative	390 (80.7)	247	143		213	177	
Positive	93 (19.3)	58	35		50	43	
Differentiation				0.021			0.038
Well	71 (14.7)	42	29		38	33	
Moderate	323 (66.9)	217	106		187	136	
Poor	89 (18.4)	46	43		38	51	
Depth of invasion				<0.001			0.003
T1	87 (18.0)	81	6		63	24	
T2	80 (16.6)	54	26		41	39	
T3	265 (54.9)	154	111		134	131	
T4	51 (10.5)	16	35		25	26	
Nodal metastasis				<0.001			<0.001
Negative	274 (56.7)	199	75		169	105	
Positive	209 (43.3)	106	103		94	115	

### Prognostic factors

Univariate analyses showed that tumor length, differentiation, vessel involvement, perineural invasion, depth of invasion, nodal metastasis, NLR and PLR were predictive of survival. Then multivariate analyses were performed with the Cox proportional hazards model. In that model, we demonstrated that differentiation (*P* = 0.015), depth of invasion (*P* = 0.024), nodal metastasis (*P* < 0.001), NLR (*P* = 0.039) and PLR (*P* < 0.001) were independent prognostic factors (Table 2). However, our study demonstrated a better discrimination for the PLR in terms of HR than the NLR (HR = 1.840 *versus* HR = 1.339, respectively).

**Table 2:** Univariate and multivariate analyses in 483 patients with ESCC

	**Survival (%)**	**Univariate analysis HR (95% CI)**	***P*-value**	**Multivariate analysis HR (95% CI)**	***P*-value**
Age (years)			0.780		0.508
≤60	49.5	1.000		1.000	
>60	49.5	1.075 (0.647-1.785)		1.092 (0.841-1.420)	
Gender			0.071		0.384
Female	61.1	1.000		1.000	
Male	47.4	1.437 (0.969-2.131)		1.197 (0.798-1.795)	
Tumor location			0.069		0.189
Upper/Middle	53.6	1.000		1.000	
Lower	44.0	1.262 (0.982-1.623)		1.187 (0.915-1.539)	
Tumor length (cm)			<0.001		0.561
≤3	66.7	1.000		1.000	
>3	42.6	2.158 (1.564-2.977)		1.116 (0.771-1.616)	
Vessel involvement			0.001		0.607
Negative	52.6	1.000		1.000	
Positive	32.9	1.673 (1.228-2.279)			
				1.088 (0.788-1.502)	
Perineural invasion			0.028		0.331
Negative	51.8	1.000		1.000	
Positive	39.8	1.397 (1.036-1.884)		1.168 (0.854-1.598)	
Differentiation			0.008		0.015
Well/Moderate	51.9	1.000		1.000	
Poor	38.6	1.507 (1.114-2.040)		1.481 (1.081-2.030)	
Depth of invasion			<0.001		0.024
T1-2	68.0	1.000		1.000	
T3-4	39.5	2.363 (1.746-3.199)		1.521 (1.056-2.191)	
Nodal metastasis			<0.001		<0.001
Negative	65.0	1.000		1.000	
Positive	29.2	2.795 (2.158-3.621)		2.105 (1.586-2.795)	
NLR			<0.001		0.039
<3.5	57.7	1.000		1.000	
≥3.5	35.4	1.947 (1.513-2.506)		1.339 (1.015-1.768)	
PLR			<0.001		<0.001
<150	63.5	1.000		1.000	
≥150	32.7	2.245 (1.735-2.904)		1.840 (1.407-2.407)	

CI, confidence interval; ESCC, esophageal squamouscell carcinoma; HR, hazard ratio; NLR, neutrophil lymphocyte ratio; PLR, platelet lymphocyte ratio.

### Overall survival

**Figure 2:**
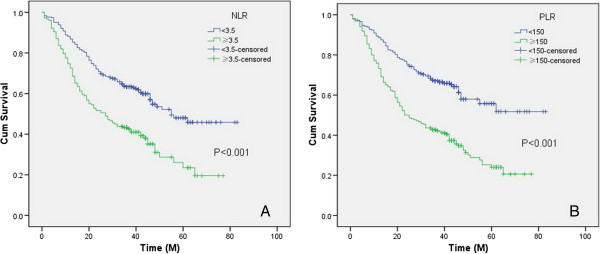
**The overall survival grouped by NLR and PLR.** Patients with NLR ≥3.5 had significantly poorer overall survival compared to NLR <3.5 (35.4% *versus* 57.7%, *P* < 0.001) **(A)**. Patients with PLR ≥150 also had significantly poorer overall survival compared to patients with PLR <150 (32.7% *versus* 63.5%, *P* < 0.001) **(B)**. NLR, neutrophil lymphocyte ratio; PLR, platelet lymphocyte ratio.

The overall survival was 49.5% in our study. Patients with NLR ≥3.5 had significantly poorer overall survival compared to NLR <3.5 (35.4% *versus* 57.7%, *P* < 0.001) (Figure 2A). Patients with PLR ≥150 also had significantly poorer overall survival compared to patients with PLR <150 (32.7% *versus* 63.5%, *P* < 0.001) (Figure 2B).

### ROC curve for overall survival prediction

**Figure 3:**
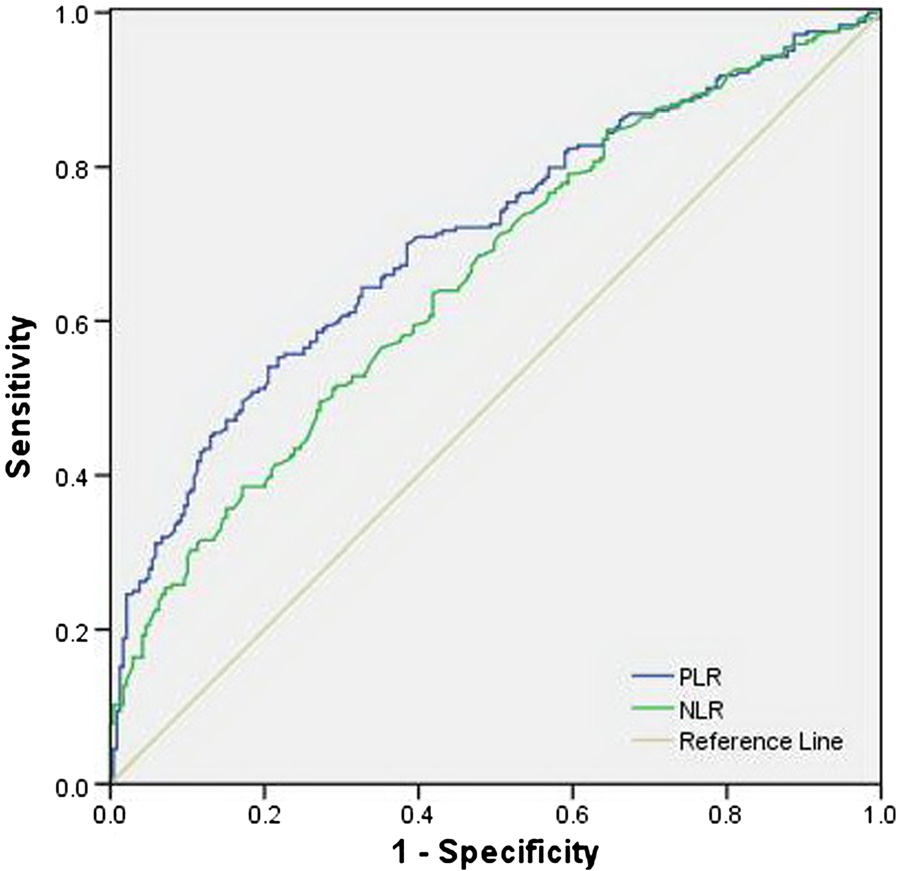
**The ROC curves grouped by NLR and PLR.** The ROC for NLR is represented by the green line with an AUC = 65.8% with a sensitivity of 49.6% and a specificity of 72.8%, and the ROC for PLR is represented by the blue line with an AUC = 70.8% with a sensitivity of 54.1% and a specificity of 79.5%. AUC, area under the curve; NLR, neutrophil lymphocyte ratio; PLR, platelet lymphocyte ratio; ROC, receiver operating characteristic.

The AUC was 0.658 (95% CI*:* 0.610 to 0.706, *P* < 0.001) for NLR and 0.708 (95% CI*:* 0.662 to 0.754, *P* < 0.001) for PLR, indicating that PLR was superior to NLR as a predictive factor in patients with ESCC (Figure 3).

## Discussion

To the best of our knowledge, this is one of the largest studies to evaluate the value of NLR and PLR in predicting prognosis for patients with ESCC. In addition, this is the first study to show PLR as an independent prognostic factor in patients with ESCC. Our study showed that high preoperative NLR (≥3.5 *versus* < 3.5, *P* = 0.039) and PLR (≥150 *versus* < 150, *P* < 0.001) were significantly associated with poor overall survival in multivariate analysis. However, our study demonstrated a better discrimination for the PLR in terms of HR than the NLR (HR = 1.840 *versus* HR = 1.339, respectively). The AUC was 0.658 for NLR and 0.708 for PLR, indicating that PLR was superior to NLR as a predictive factor in patients with ESCC.

There is a strong linkage between inflammation and cancer [7,8]. Systemic chemotherapy or radiation will inevitably have an impact on the systemic inflammation. Thus, evaluation of NLR and PLR in neoadjuvant or adjuvantchemoradiotherapy does not reflect the baseline impact of systemic inflammation on clinical outcome in EC patients. Therefore, in our study,we evaluate the potential prognostic role of preoperative NLR and PLR in patients undergoing esophagectomy for ESCC without neoadjuvant or adjuvant treatment.

Cancer-related inflammation causes suppression of antitumor immunity by recruiting regulatory T cells and activating chemokines, which results in tumor growth and metastasis. The presence of both neutrophilia and thrombocytosis tends to represent a nonspecific response to cancer-related inflammation [14]. The mechanism between preoperative leukocytosis and neutrophilia and cancer remains unclear. However, cancer has been shown to produce myeloid growth factors, such as granulocyte colony-stimulating factor, tumor necrosis factor-alpha, interleukin-1, and interleukin-6, which may influence tumor-related leukocytosis and neutrophilia [15,16].

Preoperative NLR is inversely related to prognosis in many cancers; however, its role in EC is still controversial. Sato *et al*. [10] and Sharaiha *et al*. [11] demonstrated that a high NLR is associated with tumor progression and poor survival in patients with EC. However, Dutta *et al*. [17] and Rashid *et al*. [13] showed that NLR does not correlate with prognostic factors in EC. However, there have been few studies regarding PLR in EC patients. Dutta *et al*. [17] showed that PLR does not correlate with prognostic factors in patients with EC. In our study, however, high preoperative NLR (≥3.5 *versus* < 3.5, *P* = 0.039) and PLR (≥150 *versus* < 150, *P* < 0.001) were significantly associated with poor overall survival in multivariate analysis. In addition, our study also demonstrated a better discrimination for the PLR in terms of HR than the NLR (HR = 1.840 *versus* HR = 1.339, respectively).

In the present study, the correlation between NLR and PLR was determined. As expected, we found that there was a positive correlation between the NLR and PLR (r = 0.483, *P* < 0.001). Finally, ROC curves were also plotted to verify the accuracy of NLR and PLR for survival prediction. The AUC was 0.658 (95% CI: 0.610 to 0.706, *P* < 0.001) for NLR and 0.708 (95% CI: 0.662 to 0.754, *P* < 0.001) for PLR, indicating that PLR was superior to NLR as a predictive factor in patients with ESCC.

The potential limitations of the present study include the use of a retrospective analysis and the short duration of the mean follow-up duration. In addition, because the study used data from a single institution but with different pathologists and different surgeons, there may have been a lack of uniformity. Furthermore, we excluded patients who had adjuvant chemotherapy and/or radiotherapy, which may have influenced our analysis. Thus, larger prospective studies will need to be performed to confirm these preliminary results.

## Conclusions

In summary, our study showed that preoperative NLR and PLR are significant predictors of overall survival in patients with ESCC. However, PLR is superior to NLR as a predictive factor in patients with ESCC. Thus, larger prospective studies will need to be performed to confirm these preliminary results.

## Competing interests

The authors declare that they have no competing interests.

## Authors’ contributions

JFF conceived this study, collected data, performed the analysis and drafted the manuscript. YH participated in study design, literature search and coordination. JFF and YH performed data analysis and helped to draft the manuscript. QXC participated in study design and helped to draft the manuscript. All authors read and approved the final manuscript.
